# Assessment of health-related quality of life using the SF-36 in Chinese cervical spondylotic myelopathy patients after surgery and its consistency with neurological function assessment: a cohort study

**DOI:** 10.1186/s12955-015-0237-1

**Published:** 2015-03-26

**Authors:** Yilong Zhang, Feifei Zhou, Yu Sun

**Affiliations:** Department of Orthopedics, Peking University Third Hospital, Beijing, 100191 China

**Keywords:** Cervical spondylotic myelopathy, Quality of life, Neurological dysfunction

## Abstract

**Background:**

We aimed to calculate the responsiveness and statistically prove the reliability of the Medical Outcomes Study Short Form Health Survey (SF-36) in a prospective cohort study. We investigated the profile of mid-term health-related quality of life (QOL) outcome assessments after surgery for cervical spondylotic myelopathy (CSM) and determined the consistency of the SF-36 assessments of neurological function.

**Methods:**

A total of 142 consecutive patients with CSM who underwent surgery were enrolled in the study. QOL and neurological assessments were evaluated before and at 3 months, 1 year, and more than 2 years postoperatively. We subsequently analyzed the reliability and responsiveness of the SF-36 and the QOL profile for its consistency regarding the neurological function assessment.

**Results:**

(1) Cronbach’s α ranged from 0.73 (for role-emotional) to 0.85 (for physical function). The effect size ranged from 0.57 to 0.93 for SF-36’s eight scales. Minimum clinically important differences (MCIDs) in the physical scores (PCS) and mental scores (MCS) were 5.52 and 3.43, respectively. (2) The scores for all SF-36 scale sections indicated that patients with CSM were significantly impaired compared with healthy adults. SF-36 PCS and MCS peaked at 17.7 and 18.9 months after surgery, respectively. (3) At 3 months after surgery, improvements in the modified Japanese Orthopaedic Association (mJOA) scores significantly correlated only with patients’ physical function and bodily pain scores. At 1 year after surgery, improvements in the mJOA scores significantly correlated with physical function, general health, social function, and role-emotional. At the final follow-up, improvements in the mJOA scores significantly correlated with physical function, vitality, and role-emotional.

**Conclusions:**

SF-36 is reliable and has moderate responsiveness for evaluating patients with CSM, with MCID at 5.52 for the PCS and at 3.43 for the MCS. The preoperative QOL of the CSM patients was severely impaired compared with that of the normal population. Postoperatively, each SF-36 domain improved to a variable degree. During the early stage of recovery the mJOA score improvements correlated with SF-36’s physical component domains, whereas during the later stages the improvements were associated with the mental component domains.

## Background

The Medical Outcomes Study Short Form Health Survey (SF-36) is a health status questionnaire that was developed approximately two decades ago to assess functional status and well-being [1]. The SF-36 has been applied in a variety of clinical settings, including orthopedic surgery, for which it has frequently been used to evaluate the psychometric and clinimetric properties of other self-report questionnaires [2-6].

Cervical spondylotic myelopathy (CSM) is one of the most prevalent and increasingly observed neurological disorders in the geriatric population, and surgical management has been considered the most effective treatment approach [7,8]. The time required to attain maximum recovery after surgery is one of the greatest concerns of both physicians and patients. Little relevant information, however, has been reported on the subject. To date, few studies have focused on SF-36 scores after surgery for CSM patients or on their consistency with neurological function assessments. To fill this void, we designed the current study to involve the collection of prospective patient data and the retrospective analysis of follow-up data obtained from 142 patients with CSM who underwent surgical treatment. We then calculated the responsiveness and statistically demonstrated the reliability of the SF-36 in a cohort of Chinese patients. We subsequently explored the profiles of mid-term health-related quality of life (QOL) outcome assessments after surgery for these CSM patients and their consistency with neurological function assessments using a prospective cohort study.

## Methods

### Study design and population

We prospectively collected data for 142 CSM patients who were admitted and treated at our institution from February 2008 to November 2011.We used the modified Japanese Orthopaedic Association (mJOA) assessment and the SF-36 to evaluate the patients preoperatively and again at follow-up visits at 3 months, 1 year, and more than 2 years after their surgery. The patients were diagnosed with CSM based on their disease history, physical examination, imaging studies, and having undergone ineffective conservative treatment. Patients who experienced trauma during the same period or who had had poorly controlled disorders preoperatively (e.g., serious systemic infection, coronary heart disease, diabetes) were not included in this study. The same group of surgeons in our hospital treated the enrolled patients. We then analyzed the reliability and responsiveness of the SF-36 and QOL profile, and their consistency with neurological function assessments.

### Treatment efficacy evaluation methods

The patients completed all of the evaluations under the guidance of specially trained medical personnel. All follow-up evaluations were performed at an outpatient clinic.

- mJOA assessment: This evaluation method, released by the Japanese Orthopaedic Association (JOA) on March 18, 1994, after several amendments, is used to evaluate spinal cord function of patients with CSM. The scores range from 0 to 17. The items evaluated are upper and lower extremity motor functions, sensory disturbances, and bladder function [9].
- Health measurement scale (SF-36): The SF-36 is a concise health measurement scale that was developed by the American Institute of Health (in Boston, MA). From a quantitative perspective, this scale provides a more intuitive and comprehensive reflection of the health status of a population. The scale covers eight aspects of health-related QOL: physical function (PF), role-physical (RP), bodily pain (BP), general health (GH), vitality (VT), social function (SF), role-emotional (RE), and mental health (MH). For comparison, these eight sections can be categorized into two areas: SF-36 physical scores (PCS) and SF-36 mental scores (MCS). The health transition item (HTI), derived from the HTI of the SF-36, refers to how the patient feels at the time of the questionnaire compared with 1 year prior [10].
- Peak recovery time: This measure was determined as the time point at which maximum recovery was obtained. It was evaluated for each patient. When the curve of the patient’s scores displayed double peaks, the earlier time point was considered the peak time.

### Statistical analysis

The SPSS 13.0 statistical package (SPSS, Chicago, IL) was used to establish a database and perform data management and analysis. The means and standard deviations (SDs) were computed for continuous variables. The analysis methods included descriptive statistics and numerical data. The prevalences of the lowest scores, 0/100 (floor effect), and highest scores, 100/100 (ceiling effect), for the PCS and MCS of the SF-36 were calculated. Intergroup comparisons were conducted using the Mann–Whitney U-test. Correlation analyses were performed using Spearman’s rank correlation analysis. The reliability and internal consistency of the SF-36 were assessed via Cronbach’s α and the Nunnally criterion of 0.7 [11]. The responsiveness to change in the PCS and MCS were assessed using effect sizes (ESs) and minimum clinically important differences (MCIDs). The ES was calculated by dividing the change in the score for each measure by its baseline SD [12]. The ESs were categorized as small (0.20–0.49), medium (0.50–0.79), or large (≥0.8), according to Cohen’s classification [13]. The MCID was defined as the smallest difference in a score that the patients perceived as beneficial and that could mandate a change in their management [14]. We used a distribution-based method to calculate the MCID values for both QOL instruments and applied the following formula: 1-SEM [a change of 1 standard error of the mean (SEM)] = SD × √(1-α), where α is the Cronbach’s reliability coefficient [15]. A change of 1 SEM was empirically demonstrated to correspond to the MCID in a previous study using the SF-36, which indicates that the 1-SEM criterion can be applied as a proxy for a clinically meaningful change. Values less than p < 0.05 were considered statistically significant.

## Results

Altogether, 142 patients with CSM who were treated by the same group of spinal surgeons in our hospital from February 2008 to November 2011 were enrolled in the study. The group comprised 84 men and 58 women whose ages ranged from 32 to 90 years (mean 60.0 years). The mJOA follow-up rates were 93.0% (132/142) at 3 months after surgery, 84.5% (120/142) at 1 year after surgery, and 99.3% (141/142) at the final follow-up more than 2 years postoperatively. The SF-36 follow-up rates were 88.7% (126/142) at 3 months, 84.5% (120/142) at 1 year, and 98.6% (140/142) at the final follow-up.

Regarding the current surgery index, 35.9% (51/142) of the patients underwent anterior cervical discectomy with fusion, 4.9% (7/142) underwent anterior cervical corpectomy with fusion, 7.9% (10/142) underwent artificial intervertebral disc replacement, and 52.1% (74/142) underwent laminoplasty. The mJOA scores are shown in Table 1.

**Table 1:** **Demographics for patients with cervical spondylotic myelopathy**

**Age(years)**	**Mean = 60.0**	**Range = 32-90**	
Gender	Male = 84 (39.8%)	Female = 58 (60.2%)	
Treatment	Posterior Approach	Laminoplasty	74
	Anterior approach	ACDF	51
		Artificial Intervertebral Disk Implant	10
		ACCF	7
mJOA	Mild	82	
	Moderate	39	
	Severe	21	

ACDF: anterior cervical discectomy with fusion; ACCF: anterior cervical corpectomy with fusion; mJOA: modified Japanese Orthopaedic Association scores.

Results are the numbers of patients unless otherwise stated.

### SF-36 profile

**Figure 1:**
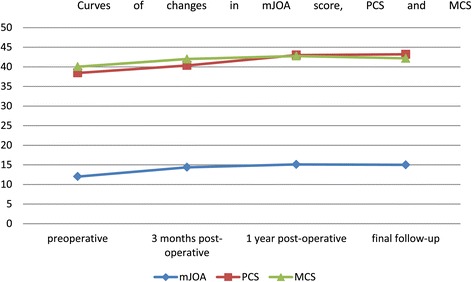
**Curves of changes in mJOAscore, PCSand MCS.**

The SF-36 reference values for a normal population were adopted from the normal reference values of populations in Hangzhou city on various subscales, including age and sex. These values were identified in 2001 by the Zhejiang University School of Medicine using the Chinese version of the SF-36 to survey the QOL of 1688 residents of Hangzhou. Prior to surgery, all patients exhibited varying degrees of decline in all sections compared with the normal population. Three sections (RP, SF, RE) exhibited the most significant declines (Table 2). At 3 months after surgery, only one section of the SF-36 (PF) had significantly improved (p < 0.05). At 1 year after surgery, five sections (PF, RP, SF, RE, MH) had significantly improved (p < 0.05). At the final follow-up, the patients exhibited significant improvements in four sections (PF, RP, SF, RE) (p < 0.05). In terms of health transition, the preoperative score of the patients was 4.09 ± 0.109. At 3 months, 1 year, and the final follow-up after posterior cervical surgery, the scores were 2.62 ± 0.142, 2.51 ± 0.118, and 2.68 ± 0.133, respectively. The PCS peaked at 17.7 months and the MCS at 18.9 months (Figure 1). Thus, during the postoperative follow-up at three time points, all three PCS scores had significantly improved compared with the preoperative score (p < 0.05). Although all three MCS scores had increased compared with the preoperative values, the differences were not significant (p > 0.05).

**Table 2:** **Comparison of preoperative and postoperative SF-36 items with the normal population**

**Parameter**	**PF**	**RP**	**BP**	**GH**	**VT**	**SF**	**RE**	**MH**
**Normal men**	81.6 ± 17.3	82.3 ± 32.2	81.8 ± 20.0	56.2 ± 20.1	55.0 ± 21.5	81.8 ± 17.6	87.1 ± 17.6	65.8 ± 17.6
**CSM men**								
Preoperation {79)	54.9 ± 24.7	16.5 ± 25.6	48.7 ± 24.4	46.5 ± 21.4	51.1 ± 19.8	51.5 ± 19.8	26.0 ± 32.2	64.3 ± 20.7
3-Months postoperation (72)	71.5 ± 23.9	21.2 ± 31.6	54.3 ± 21.8	50.9 ± 21.8	52.9 ± 19.7	56.1 ± 27.1	42.5 ± 45.9	67.3 ± 20.2
1-Year postoperation (71)	78.5 ± 19.2	40.5 ± 36.2	55.0 ± 21.8	45.4 ± 20.2	53.0 ± 20.4	63.6 ± 24.4	52.9 ± 38.5	69.7 ± 19.8
Final follow-up (83)	77.4 ± 21.6	42.2 ± 40.2	55.5 ± 25.2	49.3 ± 21.9	52.1 ± 22.9	63.2 ± 25.8	54.5 ± 43.7	66.3 ± 20.8
**Normal women**	76.8 ± 18.9	77.6 ± 36.0	74.7 ± 25.5	50.9 ± 18.4	48.0 ± 18.8	83.2 ± 19.0	87.1 ± 35.8	65.8 ± 23.3
**CSM women**								
Preoperation (53)	55.0 ± 20.7	20.6 ± 28.1	49.6 ± 21.6	45.8 ± 20.1	48.3 ± 22.3	50.3 ± 26.7	26.6 ± 29.8	60.6 ± 21.5
3-Months postoperation (54)	65.7 ± 21.1	17.6 ± 29.8	45.1 ± 17.4	47.1 ± 20.0	48.8 ± 20.2	54.2 ± 22.8	32.7 ± 39.7	66.7 ± 18.4
1-Year postoperation (49)	77.8 ± 19.3	37.8 ± 35.4	49.8 ± 17.9	43.6 ± 18.8	46.5 ± 20.1	61.2 ± 24.0	43.4 ± 38.5	64.6 ± 17.2
Final follow-up (57))	76.7 ± 21.6	37.0 ± 36.3	46.0 ± 19.8	43.8 ± 18.9	45.1 ± 21.3	58.3 ± 26.5	49.4 ± 45.8	61.6 ± 20.1

PF: physical function; RP: role-physical; BP: bodily pain; GH: general health; VT: vitality; SF: social function; RE: role-emotional; MH: mental health.

### Psychometric evaluation

The Cronbach’s α coefficient of internal consistency was used to estimate the reliability of the eight scales (Table 3). In all cases, the value exceeded the minimum standard of 0.70. Cronbach’s α ranged from 0.73 (for RE) to 0.85 (for PF).

**Table 3:** **Responsiveness, reality, and ceiling and floor effects of SF-36 scales**

**QOL measures: SF-36 scales**	**Lowest possible score (floor) (%)**	**Highest possible score (ceiling) (%)**	**Cronbach α**	**ES**
Physical function	4 (3.03)	1 (0.76)	0.85	0.91
Role-physical	13 (9.85)	3 (2.27)	0.83	0.93
Bodily pain	2 (1.51)	3 (2.27)	0.80	0.57
General health	0	1 (0.76)	0.81	0.79
Vitality	2 (1.51)	1 (0.76)	0.81	0.73
Social function	1 (0.76)	2 (1.51)	0.79	0.74
Role-emotional	21 (15.9)	7 (5.30)	0.73	0.82
Mental health	1 (0.76)	4 (3.03)	0.75	0.77

QOL: quality of life; ES: effect size.

The ceiling and floor effects identified the percentage of individual results that corresponded to the theoretical maximum or minimum (Table 3). The prevalence of patients with a ceiling and floor effect for the SF-36 scales ranged from 0 to 15.9%. The RE domain had the greatest ceiling and floor effect values.

The ES for each domain and scale was calculated, with larger values for the PF and RP subscales of the SF-36. Bodily pain demonstrated the lowest responsiveness (Table 3). ES, Cronbach’s α, and MCID of the PCS and the MCS were also calculated (Table 4).

**Table 4:** **ES, Cronbach α, MCID, and ceiling and floor effects of PCS and MCS**

**Parameter**	**PCS**	**MCS**
ES	0.84	0.81
MCID	5.52	3.43
Cronbach α	0.88	0.83
Lowest possible score (floor)	0	0
Highest possible score (ceiling)	0	0

ES: effect size; MCID: minimum clinically important difference; PCS: physical component score; MCS: mental component score.

### Consistency between objective and subjective evaluations

At 3 months after surgery, improvements in neurological function (mJOA) were significantly associated only with the PF and bodily pain of the patients (p < 0.05). At 1 year after surgery, improvements in neurological function correlated with four sections (PF, GH, SF, RE) (p < 0.05). At the final follow-up, PF, VT, and RE exhibited significant improvements in neurological function (Table 5).

**Table 5:** **Correlations between mJOA scores and various SF-36 items**

**Parameter**	**PF**	**RP**	**BP**	**GH**	**VT**	**SF**	**RE**	**MH**
CC (R) between preoperation and at 3 months after surgery (n = 116)	0.32*	0.04	0.20*	0.18	0.09	0.17	0.04	0.13
CC (R) between preoperation and at 1 year after surgery (n = 110)	0.39*	0.08	0.15	0.24*	0.13	0.22*	0.19*	0.08
CC (R) between preoperation and at final follow-up (n = 128)	0.38*	0.07	0.11	0.04	0.20*	0.16	0.20*	0.12

CC: correlation coefficient.

*p < 0.05.

## Discussion

Many researchers have reported that surgical treatment of CSM can achieve satisfactory mid-term or even long-term results [16,17]. Previously, an evaluation of the postoperative curative effects of the procedure was primarily based on alleviation of neurological dysfunction (such as that assessed by the mJOA) [18]. In recent years, however, more attention has gradually been paid to the subjective feelings of patients (assessed using SF-36) [19]. However, a comprehensive clinical assessment should consider both disease-specific and general health evaluations [20]. Few studies have focused on SF-36 scores after surgery for CSM patients or on their consistency with the assessment of neurological function. In the present study, we prospectively collected patient data and retrospectively analyzed the follow-up data of 142 patients with CSM who underwent surgical treatment. We then calculated the responsiveness and statistically demonstrated the reliability of the SF-36 in this cohort of Chinese patients. We subsequently explored the profiles of mid-term health-related QOL outcome assessments after surgery for CSM patients as well as the consistency of these profiles with neurological function assessments using a prospective cohort study.

The psychometric evaluation of the Chinese version of the SF-36 indicated that it has acceptable metric characteristics. Cronbach’s α coefficient value (all eight scales were >0.7) indicated high internal consistency of the scales. The prevalence of patients with a ceiling and floor effect for the SF-36 scales was typically less than 15%, which is considered small [21]. The RE domain had the highest ceiling and floor effect values, which is similar to the findings reported by Thakar et al. [22]. These characteristics diminished the ability of the survey to differentiate individuals according to their health status, which was clearly apparent when the results were converted to standardized or normalized values. The SF-36 was constructed to achieve the minimum standards of precision necessary for group comparisons in eight health areas. The ceiling and floor effect values for the PCS and MCS were 0. Thus, the results of the present study support the reliability and discriminative validity of the SF-36. The eight scales of the SF-36 had medium or large ES values according to Cohen’s classification, with larger values observed for PF (ES 0.91) and RP (ES 0.93), indicating high responsiveness of the SF-36. The MCIDs of the PCS and the MCS were 5.52 and 3.43, respectively.

CSM has a significant impact on QOL. The SF-36 scores indicated that, compared with the normal population, both male and female patients exhibited degrees of decline in all sections, particularly in PF, SF, and RE. The scores suggest that the impact of cervical myelopathy on patient QOL is mainly reflected in such aspects as social activities and the capacity to complete work. The health changes of the patients compared with their preoperative conditions (health transition 4.23 ± 0.692) clearly demonstrated a significant impact on QOL.

The HTI scores of the patients at 3 months after surgery, 1 year after surgery, and the final follow-up more than 2 years after surgery were 2.14 ± 0.741, 2.41 ± 0.622, and 2.62 ± 0.564, respectively, which demonstrates that surgical treatment has a positive role in the subjective evaluation of patients with CSM [23,24]. Al-Tamimi et al. reported similar results in 2013 [25]. At 3 months after surgery, the SF-36 scores of the patients exhibited significant improvement in only one item (PF), which was not consistent with the significantly improved neurological function observed after surgery. A potential explanation for this result might be that at 3 months after surgery, despite improved neurological function and somewhat improved sensory and motor functions compared with the preoperative conditions, factors such as postoperative wound pain and wound-related restrictions on activities prevented direct reflection of improved neurological function in the patients [26-29]. As wounds healed and the capacity and intent of the patients to participate in social activities increased, the patients exhibited significant improvements in PF, RP, SF, and emotional function at the mid-term and long-term follow-up evaluations compared with the preoperative conditions. As a result, an improvement in neurological function could play a meaningful role in the daily activities of the patients. Prior to surgery, the patients might even have expected improvements in these four aspects. Not all of the SF-36 scales had improved, however, which is inconsistent with the results reported by Thakar [30].

At 3 months after surgery, the neurological improvement mainly exhibited high correlations with the PF section on the SF-36 scale of the subjective evaluation. Patients considered that physical functions (e.g., walking up and down stairs, bathing, getting dressed, completing normal work) had improved with enhanced neurological function. At the later recovery time points (e.g., 1 year after surgery), the SF-36 scale sections that correlated with improvements in the mJOA scores included both physical and mental functions. At the final follow-up, however, the mJOA score was more highly associated with the mental function sections, indicating that improvements in neurological function would ultimately lead to improved mental function in patients over a longer period of time.

Figure 1 shows that the mJOA scores, PCS, and MCS reached their maximum values at 16.4, 17.7, and 18.92 months after surgery, respectively. The results reported by Suzuki et al. in 2009 demonstrated that the mJOA score peaked at 8.7 months after surgery and that the recovery process could persist for up to 2 years [31]. The results of the present study, however, indicated that the mJOA score peaked at 16.4 months after surgery and that further recovery could be expected up to 3 years postoperatively. Although the methods used in this study and that of Suzuki et al. were different, the significantly longer postoperative neurological recovery process observed for our group of patients compared with those assessed by Suzuki et al. might be a result of the differences between the Japanese and Chinese populations or between postoperative rehabilitation therapies. We believe that the data in the present study are more suitable for Chinese populations and that they provide useful information regarding the rehabilitation process for CSM patients in China. To our knowledge, there have been no reports regarding the time required after surgery to attain the maximum recovery of the PCS and MCS. The present study demonstrated the recovery process for the PCS and MCS after cervical surgery in patients with myelopathy. From another perspective, these results demonstrated that patients who first benefited from improved neurological function exhibited improvements in physiological function. Also, the psychological function improved with the gradual improvement in the physiological function. Similar to the mJOA score, the PCS and MCS recovery processes could be extended to the third year after surgery, and a small number of patients achieved their best surgical treatment results (1.77%) at 5 years postoperatively.

It is important to take note of the improvements in neurological function that did not have a positive influence on the RE and MH, such as psychological influences that affect normal work, the need to reduce normal workload or activities, and depression. Changing the medical model from a biomedical one to a bio-psycho-social structured model, and with the characteristics of CSM, reveals that the mental health of patients is of great importance.

Five patients (3.5%)—two with C5 palsy and three with cerebrospinal fluid (CSF) leak—developed complications. The development of C5 palsy negatively affects QOL regarding both the PCS and MCS. The impact is typically temporary, and over the long term the QOL is similar in patients who do and do not develop C5 palsy, which is consistent with the findings of Miller et al. [32]. When a CSF leak occurred, the leak site was dabbed with a cotton pad and the dura tear then covered with gelatin foam and sealed with fibrin glue. After the procedure, a subcutaneous drain was placed, and the skin incision was loosely approximated. The CSF leak resolved spontaneously within 3–5 days postoperatively in all three patients and did not affect the QOL of these patients, which is similar to the findings reported by Terence et al. [33].

There are some potential limitations to this study. The intervals between the follow-up time points were long, which led to relatively large errors when determining the times at which the objective and subjective rehabilitation effects reached optimal levels. Also, it was a single-center study with a mid-term follow-up analysis. Multicenter and long-term follow-up studies are needed to analyze further the subjective and objective postoperative rehabilitation patterns of patients with CSM.

## Conclusions

The SF-36 is a reliable instrument for evaluating patients with CSM, demonstrating MCIDs of 5.52 for the PCS and 3.43 for the MCS. Compared with the normal population, patients with CSM clearly have worse QOL prior to surgery. Each domain of the SF-36 improved to some degree. Early improvements in the objective evaluations based on the mJOA scores indicated a high correlation with the PF section of the SF-36 scale for the subjective evaluation. With extended recovery, the sections of the SF-36 scale that were correlated with improvements in the mJOA scores encompassed both physical and mental functions. Improvements in neurological function, however, were primarily correlated with the mental function section.
